# 3D Comparison of Mandibular Response to Functional Appliances: Balters Bionator versus Sander Bite Jumping

**DOI:** 10.1155/2018/2568235

**Published:** 2018-04-24

**Authors:** Francesca Gazzani, Antonio Carlos de Oliveira Ruellas, Kurt Faltin, Lorenzo Franchi, Paola Cozza, Renato Bigliazzi, Lucia Helena Soares Cevidanes, Roberta Lione

**Affiliations:** ^1^Department of Clinical Sciences and Translational Medicine, University of Rome “Tor Vergata”, Rome, Italy; ^2^Federal University of Rio de Janeiro, Rio de Janeiro, RJ, Brazil; ^3^School of Dentistry, University of Michigan, Ann Arbor, MI, USA; ^4^Department of Orthodontics, School of Dentistry, University Paulista, São Paulo, SP, Brazil; ^5^Department of Surgery and Translational Medicine, University of Florence, Florence, Italy; ^6^Department of Orthodontics and Pediatric Dentistry, School of Dentistry, University of Michigan, Ann Arbor, MI, USA; ^7^Department of Dentistry, UNSBC, Tirana, Albania; ^8^Department of Pediatric and Social Dentistry, Dental School of Araçatuba, Universidade Estadual Paulista (UNESP), Araçatuba, SP, Brazil

## Abstract

**Aim:**

To assess the three-dimensional (3D) maxillomandibular and dental response to Balters Bionator (BB) and the Sander Bite Jumping Appliance (SBJA) in growing patients.

**Materials and Methods:**

Twenty-seven Class II division 1 patients (13 males, 14 females), consecutively treated with either the BB (9 females, 7 males; 10.1 ± 1.6 years) or SBJA (5 females, 6 males; 11 ± 1.9 years), were collected from a single orthodontic practice. All patients presented overjet ≥5 mm, full Class II or end-to-end molar relationship, mandibular retrusion. CBCT scans were available at T1 and after removal of the functional appliances (T2) with a mean interval of 18 months. The 3D location and direction of skeletal and dental changes with growth and treatment were quantitatively assessed. Statistical analysis was performed by means of Mann–Whitney *U* test (*p* < 0.05).

**Results:**

Patients treated with the SBJA and BB orthopedic appliances presented, respectively, 4.7 mm and 4.5 mm of 3D displacement of the chin, with marked ramus growth of, respectively, 3.7 mm and 2.3 mm. While the mandible and maxilla grew downward and forward, no opening of the mandible plane was observed. Both appliances adequately controlled labial inclination of lower incisors (1.3° and 0.3°, for the SBJA and BB groups, resp.). No significant between-group differences were found for the T2−T1 changes for any of the variables, with the exception of molar displacements (significantly greater in the SBJA group than in the BB group, 1.2 mm and 0.9 mm, resp.).

**Conclusions:**

The maxillomandibular and dental growth responses to BB and SBJA therapies are characterized by vertical ramus growth and elongation of mandible that improve the maxillomandibular relationship with adequate control of lower incisor position.

## 1. Introduction

Class II division 1 malocclusion can have discrepancies in all three dimensions in the form of narrow maxilla, high palate, and sagittal discrepancy. Mandibular skeletal retrusion is commonly associated with a Class II malocclusion [1]. Functional treatment stimulates mandibular growth by forward posturing of the mandible with the condyles displaced downward and forward in the glenoid fossa [1, 2]. Due to the relative simplicity in the construction and in the clinical handling, the Balters Bionator (BB) is one of the most commonly used appliances [2]. Several investigations [3, 4] analyzed the dentoskeletal effects of the Bionator reporting a favorable increase in total mandibular length maintained in the long-term. Other functional appliances were developed such as the Sander Bite Jumping Appliance (SBJA). The appliance consists of an upper and lower unit and positions the mandible forward through the use of two prongs. Few studies [5, 6] investigated the effects of the SBJA. Martina et al. [5] reported a significant increase in mandibular length, reduced dental overjet, and the correction of molar relationship. Previous investigations using two-dimensional data have not elucidated the complex dental and skeletal components in facial growth and response to treatment that grow with different timing relative to each other. Three-dimensional (3D) Cone Beam Computed Tomography (CBCT) data has overcome these inadequacies by measuring key maxillary and mandibular adaptive and positional changes relative to the anterior cranial base [7–10].

Hence, the aim of the present investigation was to assess the three-dimensional (3D) maxillomandibular and dental response to BB and SBJA therapies in growing Class II patients.

## 2. Materials and Methods

Sample size determination revealed that, for the independent sample *t*-test, for a clinically significant difference 2.5 mm for Pogonion displacement (primary endpoint), a SD of 1.8 mm [8], an alpha level of 0.05, a power of 0.8, and a minimum of 10 subjects in each group were required (SigmaStat 3.5, Systat Software, Point Richmond, CA).

The treated group was collected from a single orthodontic practice and consisted of 27 Class II division 1 patients (13 males, 14 females), consecutively treated with either the BB (16 subjects; 9 females, 7 males; 10.1 ± 1.6 years) or SBJA (11 subjects; 5 females, 6 males; 11 ± 1.9 years). The study project was approved by the Ethical Committee at the Universidade Estadual Paulista (UNESP), Dental School of Araçatuba (protocol number 1.521.723), and informed consent was obtained from the subjects' parents.

All patients showed the following dentoskeletal features before therapy (T1): overjet greater than 5 mm (5 ≤ OVJ ≤ 8 mm), full Class II or end-to-end molar relationship, mandibular retrusion determined by cephalometric analysis of Ricketts et al. [11] and Schwarz, modified by Faltin Jr. et al. [12]. In order to evaluate skeletal maturity before (T1) and after treatment (T2), cervical vertebral maturation (CVM) method was used [13] by an operator (L.F.) calibrated in this method, by extracting a ceph image from 3D images.

In the BB group, 3 patients were at CS1, 4 patients were at CS2, 8 patients were at CS3, and 1 patient was at CS4 before treatment. The SBJA group consisted of 2 patients at CS1, 4 patients at CS2, 3 patients at CS3, and 2 patients at CS4. No permanent teeth were congenitally missing or extracted before or during treatment. The demographic data of the TGs are reported in Table 1.

The treatment protocols consisted either of a BB constructed without coverage of the lower incisors as described by Antunes et al. [3] or of a SBJA attached to the upper and lower arches by circumferential clasps without capping of the upper and lower incisors, lower and upper central screws activated only in the presence of constricted arches. The upper expansion screw is molded with two robust 13 mm long prongs, embedded in the upper plate, and positioned to form an angle ranges from 60° ± 5° with reference to the occlusal plane and according to the facial type. The mid-portion of the lower plate has an inclined plane made of acrylic, which meets with the upper prongs, so that the patient is forced to posture the mandible forward. Both appliances were fabricated using a construction bite that positioned the mandible anteriorly in an edge-to-edge incisor relationship allowing the complete disocclusion of posterior teeth. In both cases, the construction bite did not exceed 4 mm of mandibular advancement. All patients were instructed to wear the devices 16–18 hours per day. Patient compliance and the success of therapy in terms of correction of Class II malocclusion were not an inclusion criterion, so that sample selection was conducted irrespective of clinical results.

No new CBCTs were acquired for the present retrospective study. For each subject CBCT scans were already available at T1 and immediately after removal of the functional appliances (T2) with a mean interval of 18 months between the two observation times (BB 18 ± 3 m, SBJA 17.5 ± 2 m). The scans were taken using the i-CAT Vision (Imaging Sciences International, PA, USA) with a 40-second scan and a 13 × 17 cm field of view. The indication of CBCT scans was performed in accordance with the “as-low-as-reasonably-achievable” (ALARA) principle, where the radiation dose for all patients was optimized to achieve the lowest practical level to address the clinical situation. Every precaution was taken to reduce radiation dose and ensure the patient's safety during CBCT imaging. Recent improvements in reconstruction algorithms in new CBCT machines have highly decreased the necessary dose to obtain good resolution images. Diagnostic uses of CBCT, as recommended by the American Association of Oral Maxillofacial Radiology, include skeletal Class II malocclusions. Dosimetry for standard CBCT exposure settings demonstrated significant reductions in effective dose associated with the use of small FOV sizes [14, 15].

**Figure 1:**
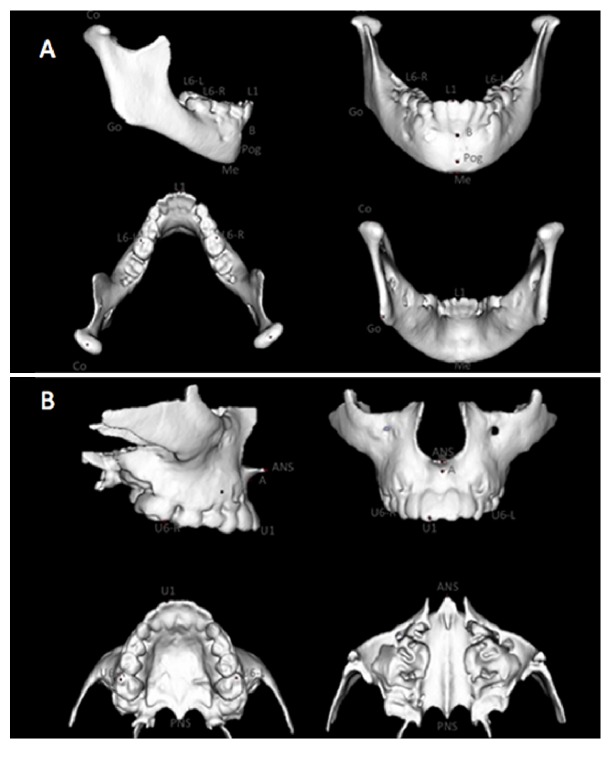
(A) 3D mandibular landmarks: Co (Condilion), Go (Gonion), Me (Menton), Pog (Pogonion), B (point B), L1 (lower incisor), L6-L (lower left molar), and L6-R (lower right molar). Mandibular Measurements: Co-Pog middle point T1, T2, T2−T1 (mandibular lenght); Co-Go middle point T1, T2, T2−T1 (ramus height); Pg T2−T1 (Pog point displacement); GoMeT2^∧^GoMeT1 (mandibular plane); L1 T2−T1 (lower incisor displacement); L1-axis T2^∧^T1 (lower incisor inclination); L6 T2−T1 (L6 displacement). (B) 3D maxillary landmarks: SNA (anterior nasal spine), PNS (posterior nasal spine), A (Point A), U1 (upper incisor), U6-L (upper left molar), and U6-R (upper right molar). Maxillary measurements: point A T1-T2 (A point displacement); PNS-ANS T1^∧^T2 (palatal plane); U1 T1-T2 (displacement); U1-axis T1^∧^T2 (upper incisor inclination); U6 T1-T2 (U6 displacement).

Skeletal and dental landmarks were placed on the greyscale voxel using ITK-SNAP (open source software, http://www.itksnap.org). Three-dimensional landmark location was verified in the axial, sagittal, and coronal multiplanar slices as well as in the 3D surface model. Assessment of starting forms was performed using point-to-point landmarks measurements (Slicer open source software, http://www.slicer.org). ITK-SNAP was used to construct virtual 3D surface models. Scans at T1 and T2 were registered on the anterior cranial base using a fully automated voxel-wise rigid registration technique described by Cevidanes et al. [8, 16, 17]. Quantitative evaluations of growth and treatment response were calculated using 3D distances and angles and their vectorial components in the anteroposterior, superior-inferior, and right-left direction. Qualitative changes were graphically displayed with color-coded distance maps. The reference points used are shown in Figure 1.

To illustrate the mandible growth at the condyles and ramus, regional superimposition of the T1 and T2 mandibles was used. For 3D mandibular regional superimposition, the region of reference included the body of mandible. To properly identify the direction and magnitude of mandibular growth at the condyles and ramus, Spherical Harmonic algorithms (SPHARM-PDM) were applied to establish corresponding surface meshes of the superimposed mandibles. Distance magnitude and vector color-coded maps representing corresponding anatomic changes from T1 to T2 were generated in Shape Population Viewer (Slicer software).

### 2.1. Statistical Analysis

Between-group differences in chronologic age at T1 and T2 and in observation interval were tested with independent-samples *t*-tests. Differences in gender distribution and in the prevalence rates of the different stages in cervical vertebral maturation at T1 and T2 were tested with chi-squared tests (SPSS 12, SPSS Inc., Chicago, Illinois, USA).

To analyze the combined error of landmark location, tracing, and digitization error of the method, 10 randomly selected CBCT were retraced and redigitized by the same operator (F.G.) after an interval of two weeks. Wilcoxon signed-rank test was applied to evaluate the systematic error while method of moments' estimator was used to calculate the method error [18]. Descriptive statistics (mean and standard deviation) and statistical comparison were calculated for the starting forms and for the T2−T1 3D cephalometric modifications in the 2 treated groups. Independent sample *t*-tests were performed for a normal distribution of the data. The Kolmogorov-Smirnov statistics revealed that not all the variables were normally distributed, and the equality of variance was assessed using Levene's test. For the variables not normally distributed, descriptive statistics were reported as median and 25th and 75th percentiles while statistical comparisons were performed with the Mann–Whitney *U* test.

## 3. Results

No significant systematic error was found for any of the variables. The method error ranged from a minimum of 0.11 mm (*X* coordinate of upper molar displacement) to a maximum of 0.78 mm (*Y* coordinate of lower molar displacement). In Table 3, the direction of each of the directional components of the 3D measurements is indicated as follows: right, anterior, and superior are positive numbers and left, inferior, and posterior are negative values.

No significant between-group differences were found either for chronologic age at T1 and T2, for the observation intervals, for gender distribution, or for the prevalence rates of the different stages in cervical vertebral maturation. No significant between-group differences were found in any of the variables at T1 (Table 2).

**Figure 2:**
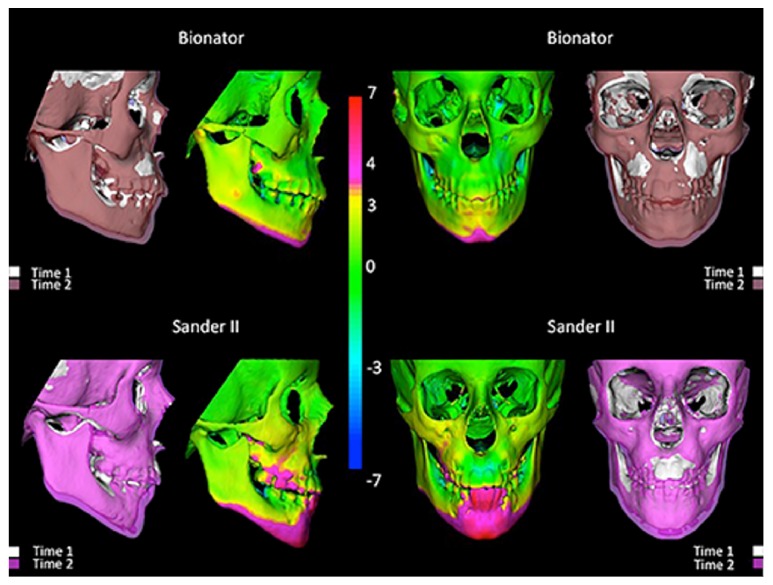
Growth and treatment changes for one patient treated with Bionator and Sander Bite Jumping. The left and right columns show the semitransparent overlays of T1 and T2 surface models superimposed on the cranial base. The center columns show color-coded closest point surface distance maps for visualization of surface distance between T1 and T2 models for each patient.

**Figure 3:**
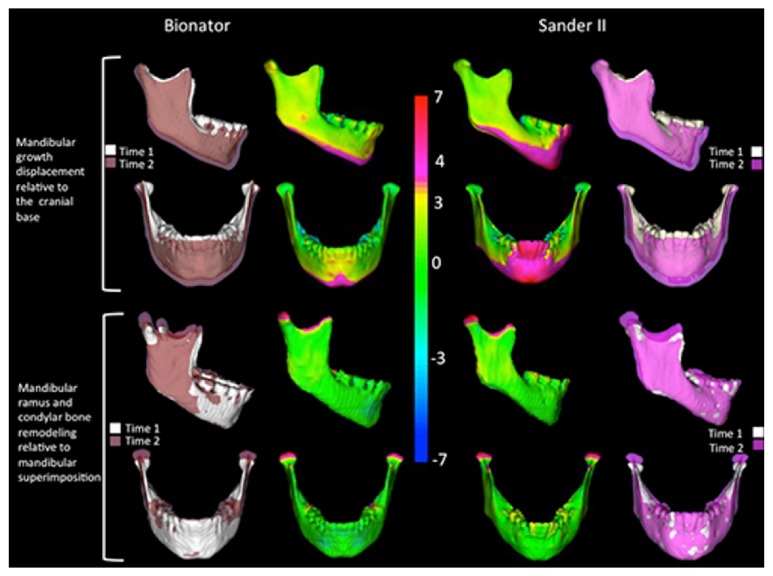
Mandibular changes relative to cranial base and regional superimposition in the mandible. The left and right columns show the semitransparent overlays of T1 and T2. The center columns show color-coded closest point surface distance maps, in millimeters, allowing visualization of surface distances between T1 and T2 models.

No significant between-group differences were found for the T2−T1 changes for any of the variables, with the exception of right-left upper and lower molar displacements that were significantly greater in the SBJA group than in the BB group (1.2 mm and 0.9 mm, resp.). Both groups presented a downward and forward maxillary and mandibular growth measured at A and Pog points. Mandibular growth was remarkably variable and predominantly vertical in a downward direction (vertical growth at Pog −3.3 mm in the SBJA group and −3.5 mm in the BB group). Forward mandibular growth was also similar in the 2 groups (1.9 mm) (Figures 2 and 3). Changes along total mandibular length (Co-Pg) also were greater in a downward direction (3.5 mm and −3.2 mm in the SBJA and BB groups, resp.) than in a forward direction (2.7 mm and 2.5 mm in the SBJA and BB groups, resp.), with marked ramus growth of, respectively, 3.7 mm and 2.3 mm. No opening of the mandible plane was observed (Table 3).

## 4. Discussion

The objective of the present study was to evaluate the 3D changes induced by BB or SBJA treatment in growing Class II patients with mandibular retrusion. No previous studies have examined the treatment outcomes of these two appliances by a thorough 3D assessment of the maxillomandibular and dental components in the correction of skeletal Class II discrepancies. With respect to conventional cephalometrics, major advantages of this method of investigation are the possibility of evaluating structures that were previously obstructed on lateral cephalograms, as well as unilateral or asymmetric anatomic modifications from growth or therapy. 3D analysis allows the clinician to rotate the 3D volumes and to observe multiple views in space rather than one sagittal view [8, 16]. The present study investigated specifically maxillary positional changes, difference in mandibular growth, and condylar and dentoalveolar modifications.

Qualitative assessment of maxillary and mandibular skeletal changes was conducted using a semitransparent overlay of superimposition and an iterative closest point distances in color-coded maps [8, 16, 17].

Subjects of both groups showed forward and downward maxillary growth at A point, as it is expected with normal growth. A maxillary restraining outcome of functional orthopedic treatment has been reported in conventional cephalometry [8] as a consequence of reciprocal force acting distally on the maxilla, when the mandible is postured forward. However, a very recent systematic review and meta-analysis [19] of controlled studies reported that, irrespective of the growth phase, no or very minimal changes were seen in terms of maxillary growth restrain [4, 20].

Interestingly, the vertical growth of the maxilla in our current study did not lead to opening of the mandibular plane angle, probably also due to the marked rami growth in both groups and the favorable differential mandibular growth.

The most important finding of the present investigation was the elongation of the mandible, with increased ramus height observed in both groups. The downward and forward mandibular displacement observed at Pog point relative to the cranial base (Table 3 and Figure 3) may be explained not only by increased mandibular length but also by the ramus growth that compensates the vertical maxillary growth with opening the mandibular plane angle. Franchi et al. [20, 21] found a significant long-term elongation of the mandible during the pubertal peak associated with an advancement of the bony chin. More recently, some authors [2, 3] compared the short-term and long-term shape and size differences in a Class II sample treated with Bionator versus an untreated Class II control group by means of the Thin-Plate Spline analysis. The mandibular forward and downward displacement was more evident at the mandibular symphysis [2, 3].

**Figure 4:**
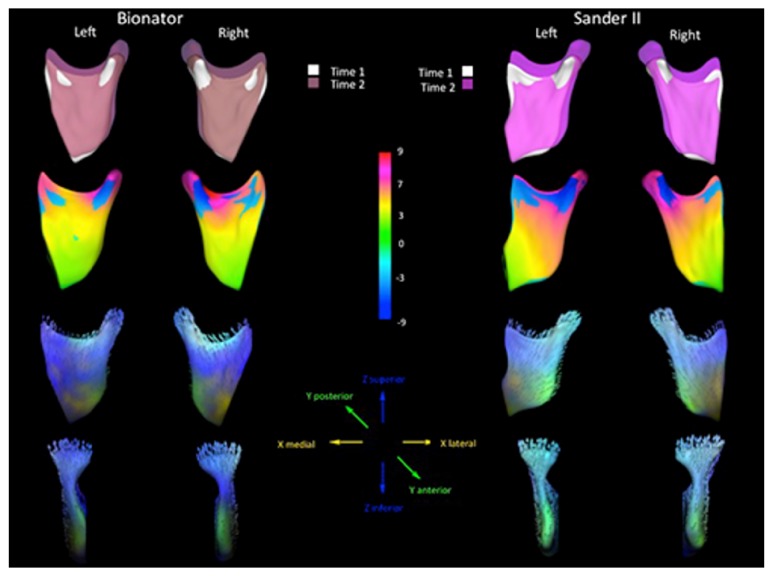
Mandibular rami and condylar changes relative to a mandibular regional superimposition. The first row shows the semitransparent overlays of T1 and T2. The central row shows color-coded corresponding surface distance maps in millimeters. Then, the last two rows show vector direction color-coded maps, allowing quantitative and qualitative evaluation of the mandibular and condylar changes.

The mandibular growth response, as observed with the mandibular regional registration, revealed remarkable individual variability and the predominantly superior and posterior growth at the condylar region (Figure 4). A more posterior and superior direction of condylar growth allows adaptation of the mandible to the skull base and a supplementary elongation. The resulting 25th and 75th percentile of increase in ramus height varied from 2.3 to 5.8 mm and 1.7 to 3.3 mm, respectively, in the SBJA and BB groups. In the present study, the favorable mandibular change was associated with the direction of condylar growth, corroborating previous studies [21, 22].

Data available in the literature about the effects determined by SBJA treatment are scarce [5, 6]. Martina et al. [5] tested the efficacy of SBJA therapy pointing out a significant increase in mandibular length and molar relationship improvement. In the present study, the SBJA group presented greater, but not statistically significant, vertical growth of the ramus compared to the BB group (Table 2 and Figure 4). Greater posterior condylar and ramus growth in patients treated with the SBJA may explain the more anterior mandibular displacement relative to the cranial base as shown in Figures 2 and 4.

Regarding the dentoalveolar effects, the SBJA induced statistically significant greater dental expansion of both upper and lower molars, as a consequence of the activation of upper and lower central screws (Table 2). Both appliances adequately controlled labial inclination of lower incisor. The absence of the coverage of the lower incisor did not affect their inclination. The Bionator produced a mild lingual inclination of the upper incisors. This effect was probably related to the lip closure with favorable negative pressure on the teeth induced by the appliance [2]. This finding is in disagreement with Lux et al. [23] who found that the correction of Class II problem with functional appliances was sustained mainly by a strong dentoalveolar component.

Limitations of the current study were its retrospective nature and the relatively small sample size, especially in the SBJA group. For this reason, the findings of the current investigation should be interpreted cautiously and need to be confirmed on a larger sample. The relative small sample size did not allow investigating the role of the individual skeletal maturity. This study's findings are also considered understanding that ethical issues regarding obtaining 3D scans of untreated Class II patients prevented us from including a Class II control group. Continued follow-up of long-term growth and treatment response of the present samples may provide further clarification of the intricate craniofacial growth and development adaptations that continue to occur until skeletal maturity.

## 5. Conclusions

This study's 3D findings elucidate how two functional orthopedic appliances effectively correct Class II malocclusion associated with mandibular deficiency. The maxillomandibular and dental growth responses to BB and SBJA therapies are characterized by vertical ramus growth and elongation of mandible that improve the maxillomandibular relationship with adequate control of lower incisor position.

## Figures and Tables

**Table 1:** Demographics of the BB and SBJA groups^a^.

Variables	SBJA (*n* = 11, 5f, 6m)		BB (*n* = 16, 9f, 7m)		*p*
	mean	SD	mean	SD	
Age T1, y	11	1.9	10.10	1.6	NS
Age T2, y	12.6	1.7	12.5	1.5	NS
T2-T1, y	1.6	0.6	1.7	0.4	NS

Descriptive statistics and statistical comparisons at T1 and T2 (independent-samples *t*-tests); ^a^T1 indicates before treatment; T2 indicates immediately after removal of the functional appliances; NS: not significant.

**Table 2:** Starting forms for the BB and SBJA groups.

Variables	SBJA group (*n* = 11; 5f, 6m)		BB (*n* = 16; 9f, 7m)		Diff.	*p* value	95% CI of the difference	
	Mean	SD	Mean	SD			Lower	Upper
Co-A (mm)	79.4	4.3	78.7	4.0	0.7	0.682	−2.646	3.979
Co-Gn (mm)	100.1	5.1	99.2	4.8	0.8	0.666	−3.137	4.828
ANB (deg.)	5.2	1.0	5.0	1.9	0.2	0.730	−1.052	1.481
Max. mand. diff. (mm)	20.7	2.6	20.5	2.5	0.2	0.860	−1.900	2.259
FH to Pal. Pl. (deg.)	−1.0	2.5	−1.2	4.0	0.2	0.878	−2.567	2.985
FH to mand. Pl. (deg.)	26.0	3.6	26.5	4.0	−0.5	0.729	−3.595	2.548
Pal. Pl. to mand Pl. (deg.)	27.3	3.3	28.6	5.7	−1.4	0.483	−5.292	2.571
ANS to Me (mm)	57.9	3.2	59.9	3.7	−1.9	0.173	−4.807	0.910
Co-Go (mm)	48.5	3.8	47.8	3.1	0.7	0.597	−2.026	3.449
Co-Go-Me (deg.)	126.0	4.3	123.7	4.8	2.3	0.211	−1.387	5.990
Upper inc. to pal. Pl. (deg.)	111.8	9.7	111.9	4.4	−0.1	0.984	−6.848	6.723
Lower inc. to mand. Pl. (deg.)	96.0	7.1	98.1	3.7	−2.1	0.376	−7.161	2.882

SD = standard deviation; Diff. = differences; CI = confidence interval; 25th/75th = 25th and 75th percentiles; deg. = degrees; Max. mand. diff. = maxillomandibular differential; FH = Frankfort horizontal; pal. = palatal; Pl. = plane; mand. = mandibular; inc. = incisor.

**Table 3:** Descriptive statistics and statistical comparisons of the T2-T1 changes of the BB and SBJA groups.

Variables	SBJA group (*n* = 11; 5f, 6m)		BB (*n* = 16; 9f, 7m)		Diff.	*p* value	95% CI of the difference	
	Mean/ median	SD 25th/75th	Mean/ median	SD 25th/75th			Lower	Upper
A point displacement T1-T2 (mm)								
Right-left	0.3	0.9	0.1	0.5	0.2	0.470	−0.4	0.8
Ant-post	1.5	1.2	1.1	1.0	0.4	0.399	−0.5	1.2
Sup-inf	−0.9	1.6	−1.9	1.6	1.0	0.122	−0.3	2.3
3D	2.5	1.1	2.6	1.3	−0.1	0.894	−1.1	0.9

Pog. point displacement T1-T2 (mm)								
Right-left	0.1	1.4	−0.3	1.0	0.4	0.459	−0.6	1.3
Ant-post	1.9	1.9	1.9	1.9	0.0	0.987	−1.5	1.5
Sup-inf	−3.5	2.3	−3.3	3.0	−0.2	0.863	−2.4	2.0
3D	4.7	1.8	4.5	2.6	0.2	0.826	−1.7	2.1

Co-Pog. middle point T2-T1 (mm)								
Right-left	0.0	1.2	−0.6	1.6	0.6	0.270	−0.5	1.8
Ant-post	2.7	1.9	2.5	1.9	0.2	0.810	−1.4	1.7
Sup-inf	−3.5	2.8	−3.2	2.6	−0.3	0.779	−2.5	1.9
3D	4.4	2.5	4.0	2.4	0.4	0.714	−1.6	2.3

Co-Go middle point T2-T1 (mm)								
Right-left	0.1	0.6	−0.4	1.2	0.5	0.205	−0.3	1.3
Ant-post	0.5	1.4	0.2	1.7	0.3	0.653	−1.0	1.6
Sup-inf	*−3.5*	−5.8/−2.3	*−2.1*	−3.3/−1.7	−1.4	0.162	−0.4	3.1
3D	*3.7*	2.3/6.0	*2.3*	1.7/3.3	1.4	0.231	−3.2	0.3

Pal plane PNS-ANS T1-T2 (deg)								
Pitch	−0.3	1.3	−0.2	1.1	−0.1	0.849	−1.1	0.9

Mand. plane GoMe T1-T2 (deg.)								
Pitch	0.5	1.6	−0.2	1.5	0.7	0.238	−0.5	2.0

U1 axis T1-T2 (deg.)								
pitch	0.5	6.0	−3.2	3.4	3.7	0.056	−0.1	7.4

U1 displacement T1-T2 (mm)								
Ant-post	0.8	1.9	0.4	1.2	0.4	0.531	−0.8	1.6
Sup-inf	−1.8	1.9	−2.8	1.4	1.0	0.118	−0.3	2.3
3D	3.0	1.4	3.2	1.3	−0.2	0.776	−1.3	1.0

U6 displacement (mm)								
Right-left	*1.3*	0.5/2.4	*0.1*	−0.2/0.5	**1.2**	**0.004**	−**1.9**	**−0.5**
Ant-post	0.6	1.1	1.0	1.2	−0.4	0.421	−1.3	0.6
Sup-inf	−1.6	1.6	−2.6	1.6	1.0	0.108	−0.2	2.3
3D	*2.3*	1.9/3.8	*2.8*	2.1/4.5	−0.5	0.473	−0.8	1.4

L1 axis T1-T2 (deg.)								
pitch	−1.3	2.6	0.3	2.9	−1.6	0.155	−3.8	0.7

U1 displacement T1-T2 (mm)								
Ant-post	2.3	1.7	1.3	1.6	1.0	0.147	−0.4	2.3
Sup-inf	−3.5	1.9	−2.9	2.3	−0.6	0.497	−2.3	1.2
3D	5.1	1.6	3.9	1.9	1.2	0.097	−0.2	2.7

L6 displacement (mm)								
Right-left	1.1	0.7	0.2	0.6	**0.9**	**0.001**	**0.4**	**1.4**
Ant-post	2.7	1.5	2.3	2.0	0.4	0.578	−1.1	1.9
Sup-inf	−3.0	1.8	−2.9	1.8	−0.1	0.910	−1.5	1.4
3D	4.6	1.8	4.1	2.2	0.5	0.599	−1.2	2.1

SD = standard deviations; Diff. = differences; CI = confidence interval; 25th/75th = 25th and 75th percentiles; deg. = degrees.
